# Supplementary material for: Adiposity QTL Adip20 decomposes into at least four loci when dissected using congenic strains
Source: PLoS One. 2017 Dec 1;12(12):e0188972. doi: 10.1371/journal.pone.0188972 (PMC5711020; doi:10.1371/journal.pone.0188972)
Supplement: S2 Table — List of mice with tumors or otherwise excluded from analysis. Weight = Body weight in grams; Length, = body length in centimeters; Gonadal = gonadal adipose depot weight at the time of dissection in grams; Tumor location includes, weights if available. (DOCX) [file pone.0188972.s002.docx]

| MouseID | Strain | Days | Weight, g | Length, cm | Gonadal, g | Tumor location |
| --- | --- | --- | --- | --- | --- | --- |
| B.2009.5051 | B6.129-Chr9 | 181 | 27.14 | 8.8 | 0.31 | Gonadal adipose depot |
| B.2011.7068 | B6.129-*Adip20* | 181 | 29.35 | 9.5 | 0.43 | Free-floating between pancreas/small intestine |
| B.2011.7313 | B6.129-*Adip20* | 181 | 30.83 | 9.6 | 0.4 | Near gonadal adipose depot |
| B.2011.7323 | B6.129-*Adip20* | 181 | 31.85 | 9.1 | 0.59 | Near gonadal adipose depot |
| B.2011.7333 | B6.129-*Adip20* | 181 | 24.97 | 8.6 | 0.14 | Near gonadal adipose depot |
| B.2011.7334 | B6.129-*Adip20* | 181 | 29.83 | 9.5 | 0.33 | Near gonadal adipose depot |
| B.2011.7918 | B6.129-*Adip20* | 180 | 36.13 | 10.1 | 1.07 | Near gonadal adipose depot |
| B.2011.8042 | B6.129-*Adip20* | 180 | 30.59 | 9 | 0.47 | Fluid filled tumor in abdomen,1.52 g |
| B.2011.8727 | B6.129-*Adip20* | 182 | 29.28 | 8.6 | 0.52 | Lungs, blood-filled |
| B.2011.8907 | B6.129-*Adip20* | 182 | 30.54 | 9.6 | 0.51 | Stomach |
| B.2011.9214 | B6.129-*Adip20* | 181 | 21.6 | 9.1 | 0.2 | Lungs |
| B.2011.9446 | B6.129-*Adip20* | 181 | 26.51 | 8.5 | 0.34 | Lungs |
| R.2011.87 | B6.129-*Adip20* | 180 | 37.49 | 9.1 | 0.85 | Tumor, unspecified |
| R.2012.1018 | B6.129-*Adip20* | 181 | 30.5 | 9.8 | 0.5 | Gonadal adipose depot,0.07g |
| R.2012.1020 | B6.129-*Adip20* | 181 | 23.82 | 8.8 | 0.21 | Large intestine, 0.26g |
| R.2012.296 | B6.129-*Adip20* | 179 | 30.28 | 9.2 | 0.51 | Gonadal adipose depot, 0.06 g |
| R.2012.330 | B6.129-*Adip20* | 181 | 31.6 | 9 | 0.39 | Tumor near right gonad |
| R.2012.490 | B6.129-*Adip20* | 182 | 24.39 | 10.4 | 0.16 | Subscapular region, bloody tumors |
| R.2012.640 | B6.129-*Adip20* | 180 | 25.8 | 9.5 | 0.18 | Lungs |
| R.2013.1260 | B6.129-*Adip20* | 181 | 30.22 | 10.1 | 0.24 | Epididymis and pancreas |
| R.2013.1263 | B6.129-*Adip20* | 183 | 35.97 | 9.2 | 0.68 | Gonadal adipose depot |
| R.2013.1527 | B6.129-*Adip20* | 180 | 31.87 | 9.4 | 0.31 | Lungs |
| R.2013.1660 | B6.129-*Adip20* | 181 | 25.93 | 9.1 | 0.13 | Testicle |
| R.2013.1805 | B6.129-*Adip20* | 103 | 30.67 | 9.2 | 0.3 | Gonadal adipose depot |
| R.2013.1939 | B6.129-*Adip20* | 180 | 30.48 | 9.1 | 0.18 | Gonadal adipose depot |
| R.2013.1940 | B6.129-*Adip20* | 180 | 31.01 | 8.9 | 0.2 | Liver |
| R.2014.2052 | B6.129-*Adip20* | 180 | 33.4 | 9.1 | 0.16 | Testicle, 0.03g |
