# Supplementary material for: Adiposity QTL Adip20 decomposes into at least four loci when dissected using congenic strains
Source: PLoS One. 2017 Dec 1;12(12):e0188972. doi: 10.1371/journal.pone.0188972 (PMC5711020; doi:10.1371/journal.pone.0188972)
Supplement: S4 Table — Weight of gonadal depot and body weight (BW) of congenic mice with donor regions of different genotypes of donor regions. aDonor region genotype: Host = B6/B6; Donor = 129/B6. bDonor region size: NA = mice without donor region; Partial = mice that inherited a partial-length (due to a recombination event) donor region from father; Full = mice that inherited full-length donor region from the father. (DOCX) [file pone.0188972.s004.docx]

| Substrain | Donor region genotype^a^ | Donor region size^b^ | N | Gonadal, g, M | Gonadal, g SD | BW, g  Mean | BW, g  SD |
| --- | --- | --- | --- | --- | --- | --- | --- |
| 1 | Host | NA | 61 | 0.51 | 0.23 | 31.6 | 3.0 |
| 1 | Donor | Partial | 33 | 0.60 | 0.23 | 32.3 | 2.8 |
| 1 | Donor | Full | 19 | 0.57 | 0.27 | 31.6 | 2.9 |
| 1.1 | Host | NA | 93 | 0.50 | 0.22 | 31.5 | 2.9 |
| 1.1 | Donor | Partial | 35 | 0.59 | 0.35 | 31.2 | 3.1 |
| 1.1 | Donor | Full | 48 | 0.62 | 0.33 | 31.6 | 3.0 |
| 1.1.1 | Host | NA | 2 | 1.09 | 0.78 | 36.6 | 6.6 |
| 1.1.1 | Donor | Full | 1 | 0.81 | NA | 35.2 | NA |
| 1.2 | Host | NA | 8 | 0.52 | 0.25 | 32.9 | 4.4 |
| 1.2 | Donor | Partial | 4 | 0.75 | 0.36 | 34.0 | 5.4 |
| 1.2 | Donor | Full | 2 | 0.73 | 0.23 | 34.4 | 2.5 |
| 3 | Host | NA | 48 | 0.52 | 0.20 | 31.2 | 3.0 |
| 3 | Donor | Partial | 97 | 0.59 | 0.27 | 31.7 | 3.3 |
| 3 | Donor | Full | 30 | 0.52 | 0.18 | 29.5 | 2.6 |
| 3.1 | Host | NA | 66 | 0.51 | 0.26 | 31.4 | 3.5 |
| 3.1 | Donor | Partial | 7 | 0.57 | 0.23 | 31.4 | 3.3 |
| 3.1 | Donor | Full | 47 | 0.60 | 0.23 | 32.0 | 2.8 |
| 3.1.1 | Host | NA | 66 | 0.49 | 0.22 | 31.6 | 3.0 |
| 3.1.1 | Donor | Partial | 6 | 0.45 | 0.15 | 33.3 | 1.4 |
| 3.1.1 | Donor | Full | 34 | 0.52 | 0.26 | 31.1 | 3.5 |
| 3.1.1.1 | Host | NA | 74 | 0.39 | 0.19 | 31.3 | 2.3 |
| 3.1.1.1 | Donor | Full | 53 | 0.38 | 0.14 | 30.8 | 2.2 |
| 3.1.1.2 | Host | NA | 19 | 0.40 | 0.19 | 31.5 | 2.9 |
| 3.1.1.2 | Donor | Full | 15 | 0.39 | 0.15 | 30.9 | 2.1 |
| 3.1.1.3 | Host | NA | 8 | 0.44 | 0.33 | 29.7 | 3.5 |
| 3.1.1.3 | Donor | Full | 6 | 0.72 | 0.41 | 33.3 | 3.3 |
| 3.1.1.4 | Host | NA | 36 | 0.33 | 0.14 | 29.6 | 2.8 |
| 3.1.1.4 | Donor | Partial | 1 | 0.43 | NA | 29.1 | NA |
| 3.1.1.4 | Donor | Full | 26 | 0.31 | 0.14 | 29.6 | 2.4 |
| 3.1.2 | Host | NA | 14 | 0.52 | 0.26 | 31.9 | 3.0 |
| 3.1.2 | Donor | Full | 7 | 0.48 | 0.21 | 31.3 | 2.1 |
| 3.1.3 | Host | NA | 6 | 0.40 | 0.14 | 30.0 | 2.5 |
| 3.1.3 | Donor | Full | 0 | NA | NA | NA | NA |
| 3.1.4 | Host | NA | 4 | 0.54 | 0.30 | 29.2 | 4.6 |
| 3.1.4 | Donor | Full | 7 | 0.86 | 0.21 | 33.3 | 2.6 |
| 3.1.4.1 | Host | NA | 12 | 0.53 | 0.24 | 33.0 | 1.6 |
| 3.1.4.1 | Donor | Partial | 1 | 0.42 | NA | 29.8 | NA |
| 3.1.4.1 | Donor | Full | 13 | 0.50 | 0.20 | 31.8 | 2.6 |
| 4 | Host | NA | 66 | 0.47 | 0.17 | 31.2 | 2.6 |
| 4 | Donor | Partial | 14 | 0.55 | 0.33 | 30.6 | 4.4 |
| 4 | Donor | Full | 39 | 0.59 | 0.24 | 31.0 | 3.2 |
| 4.1 | Host | NA | 26 | 0.44 | 0.17 | 30.6 | 2.3 |
| 4.1 | Donor | Full | 18 | 0.49 | 0.20 | 30.4 | 2.2 |
| 4.1a | Host | NA | 9 | 0.57 | 0.18 | 31.6 | 2.0 |
| 4.1a | Donor | Partial | 2 | 0.38 | 0.17 | 29.6 | 1.4 |
| 4.1a | Donor | Full | 11 | 0.49 | 0.20 | 31.4 | 2.9 |
| 4.2 | Host | NA | 8 | 0.47 | 0.18 | 30.0 | 2.6 |
| 4.2 | Donor | Full | 8 | 0.57 | 0.17 | 31.8 | 2.3 |
| 4.3 | Host | NA | 9 | 0.46 | 0.18 | 30.3 | 2.4 |
| 4.3 | Donor | Partial | 1 | 0.41 | NA | 28.5 | NA |
| 4.3 | Donor | Full | 8 | 0.59 | 0.19 | 32.3 | 2.4 |
| 4.4 | Host | NA | 32 | 0.49 | 0.30 | 31.3 | 3.0 |
| 4.4 | Donor | Partial | 1 | 0.33 | NA | 30.9 | NA |
| 4.4 | Donor | Full | 28 | 0.48 | 0.25 | 30.6 | 2.9 |
| 4.5 | Host | NA | 2 | 1.15 | 0.47 | 38.2 | 0.1 |
| 4.5 | Donor | Full | 2 | 0.65 | 0.37 | 31.6 | 4.4 |
