# Supplementary material for: Adiposity QTL Adip20 decomposes into at least four loci when dissected using congenic strains
Source: PLoS One. 2017 Dec 1;12(12):e0188972. doi: 10.1371/journal.pone.0188972 (PMC5711020; doi:10.1371/journal.pone.0188972)
Supplement: S5 Table — Genotypes and phenotypes of the 12 congenic strains (strains with numbers of mice ≥12 mice/strain, and all mice having full-length donor regions). Column headings show IDs of congenic strains. We show here only markers informative for breakpoints of the donor regions. A = B6/B6 genotype; H = 129/B6 genotype; X = data are not available. Based on analysis of gonadal depot weight (S4 and S6 Tables), we identified strains as Adip20(+) (shaded; strains with significant differences between mice with B6/B6 and 129/B6 genotypes) or Adip20(-) (no shading; strains with no significant differences between mice with B6/B6 and 129/B6 genotypes). The far right column ("Shared region") indicates whether a QTL-containing region is shared only by Adip20(+) but not by Adip20(-) strains (i.e., whether a marker genotype is H in all Adip20(+) strains and A in all Adip20(-) strains). None of the regions of chromosome 9 met this single QTL only criterion. (DOCX) [file pone.0188972.s005.docx]

| Marker | MB | 1 | 1.1 | 3 | 3.1 | 3.1.1 | 3.1.1.1 | 3.1.1.2 | 3.1.1.4 | 3.1.4.1 | 4 | 4.1 | 4.4 | Shared region | |
| --- | --- | --- | --- | --- | --- | --- | --- | --- | --- | --- | --- | --- | --- | --- | --- |
| rs3694533 | 4.02 | H | A | A | A | A | A | A | A | A | A | A | A | No |  |
| rs3719348 | 16.2 | H | A | A | A | A | A | A | A | A | A | A | A | No |  |
| rs13480087 | 18 | H | H | A | A | A | A | A | A | A | A | A | A | No |  |
| rs3675844 | 42.4 | H | H | A | A | A | A | A | A | A | A | A | A | No |  |
| rs48176249 | 42.6 | H | H | A | A | A | A | A | A | A | H | A | A | No |  |
| rs47480058 | 42.8 | H | H | A | A | A | A | A | A | A | H | A | A | No |  |
| rs4135590 | 43 | H | H | A | A | A | A | A | A | A | H | A | H | No |  |
| rs29687664 | 43.8 | H | H | A | A | A | A | A | A | A | H | A | H | No |  |
| rs32595056 | 44.1 | H | H | H | H | H | A | H | A | A | H | A | H | No |  |
| rs32600517 | 44.2 | H | H | H | H | H | A | H | A | A | H | A | H | No |  |
| D9Mit25 | 44.3 | H | H | H | H | H | A | H | A | A | H | A | H | No |  |
| rs13462199 | 44.6 | H | H | H | H | H | A | H | A | A | H | A | H | No |  |
| rs33745945 | 46.4 | H | H | H | H | H | A | H | A | A | H | A | H | No |  |
| rs3699026 | 48.8 | H | H | H | H | H | A | A | A | A | H | A | H | No |  |
| rs30353028 | 50.5 | H | H | H | H | H | A | A | A | A | H | A | A | No |  |
| D9MIT97 | 50.7 | H | H | H | H | H | A | A | A | A | H | A | A | No |  |
| D9MIT971 | 50.8 | H | H | H | H | H | A | A | A | A | H | A | A | No |  |
| rs3699358 | 51.7 | H | H | H | H | H | A | A | A | A | H | A | A | No |  |
| rs6167828 | 51.9 | H | H | H | H | H | A | A | A | A | H | A | A | No |  |
| rs3685939 | 53.5 | H | H | H | H | H | A | A | A | A | H | A | A | No |  |
| rs29645267 | 53.9 | H | H | H | H | H | A | A | H | A | H | A | A | No |  |
| rs29835751 | 54.2 | H | H | H | H | H | A | A | H | A | H | A | A | No |  |
| rs30089733 | 54.3 | H | H | H | H | H | H | A | H | A | H | A | A | No |  |
| rs225183040 | 54.3 | H | H | H | H | H | H | A | H | A | H | A | A | No |  |
| rs29736523 | 54.3 | H | H | H | H | H | H | A | H | A | H | H | A | No |  |
| rs13480208 | 55.2 | H | H | H | H | H | H | A | H | A | H | H | A | No |  |
| rs30424912 | 56.7 | H | H | H | H | H | H | A | H | A | H | H | A | No |  |
| rs3677551 | 56.7 | H | H | H | H | H | H | A | H | H | H | H | A | No |  |
| rs29785790 | 56.7 | H | H | H | H | H | H | A | H | H | H | H | A | No |  |
| rs30437080 | 56.7 | H | H | H | H | H | H | A | H | H | H | H | A | No |  |
| rs30226504 | 56.7 | H | A | H | H | H | H | A | H | H | H | H | A | No |  |
| rs29946730 | 56.7 | H | A | H | H | A | A | A | A | H | H | H | A | No |  |
| rs4227682 | 57.3 | H | A | H | H | A | A | A | A | H | H | H | A | No |  |
| D9MIT21 | 57.7 | X | A | H | H | A | A | A | A | H | H | H | A | No |  |
| rs30042362 | 57.7 | A | A | H | H | A | A | A | A | H | H | H | A | No |  |
| rs29737283 | 57.9 | A | A | H | A | A | A | A | A | A | H | H | A | No |  |
| rs4227694 | 58.3 | A | A | H | A | A | A | A | A | A | H | H | A | No |  |
| rs3685575 | 59.4 | A | A | H | A | A | A | A | A | A | A | A | A | No |  |
| rs8254399 | 124 | A | A | H | A | A | A | A | A | A | A | A | A | No |  |
