# Supplementary material for: Adiposity QTL Adip20 decomposes into at least four loci when dissected using congenic strains
Source: PLoS One. 2017 Dec 1;12(12):e0188972. doi: 10.1371/journal.pone.0188972 (PMC5711020; doi:10.1371/journal.pone.0188972)
Supplement: S7 Table — Genotypes and phenotypes of the congenic strains of any sample size, except as noted below, including mice with partial or full-length donor regions. However, we still excluded three strains from this analysis (shaded in light grey) because they had very few mice (1.1.1, 3.1.3, 4.5). For other details, see S5 Table. (DOCX) [file pone.0188972.s007.docx]

| Marker | MB | 1 | 1.1 | 1.1.1 | 1.2 | 3 | 3.1 | 3.1.1 | 3.1.1.1 | 3.1.1.2 | 3.1.1.3 | 3.1.1.4 | 3.1.2 | 3.1.4 | 3.1.3 | 3.1.4.1 | 4 | 4.1 | 4.1a | 4.2 | 4.3 | 4.4 | 4.5 | Shared |
| --- | --- | --- | --- | --- | --- | --- | --- | --- | --- | --- | --- | --- | --- | --- | --- | --- | --- | --- | --- | --- | --- | --- | --- | --- |
| rs3694533 | 4.0 | H | A | A | A | A | A | A | A | A | A | A | A | A | A | A | A | A | A | A | A | A | A | No |
| rs3719348 | 16.2 | H | A | A | A | A | A | A | A | A | A | A | A | A | A | A | A | A | A | A | A | A | A | No |
| rs13480087 | 18.0 | H | H | A | A | A | A | A | A | A | A | A | A | A | A | A | A | A | A | A | A | A | A | No |
| rs3675844 | 42.4 | H | H | A | A | A | A | A | A | A | A | A | A | A | A | A | A | A | A | A | A | A | A | No |
| rs48176249 | 42.6 | H | H | A | A | A | A | A | A | A | A | A | A | A | A | A | H | A | A | A | A | A | A | No |
| rs47480058 | 42.8 | H | H | A | A | A | A | A | A | A | A | A | A | A | A | A | H | A | A | A | A | A | A | No |
| rs4135590 | 43.0 | H | H | H | A | A | A | A | A | A | A | A | A | A | A | A | H | A | A | A | H | H | A | No |
| rs29687664 | 43.8 | H | H | H | A | A | A | A | A | A | A | A | A | A | A | A | H | A | A | A | H | H | A | No |
| rs32595056 | 44.1 | H | H | H | A | H | H | H | A | H | A | A | A | A | A | A | H | A | A | A | H | H | A | No |
| rs32600517 | 44.2 | H | H | H | A | H | H | H | A | H | A | A | A | A | A | A | H | A | A | A | H | H | A | No |
| D9Mit25 | 44.3 | H | H | H | A | H | H | H | A | H | A | A | A | X | A | A | H | A | A | A | H | H | A | No |
| rs13462199 | 44.6 | H | H | H | A | H | H | H | A | H | A | A | A | H | A | A | H | A | A | A | H | H | A | No |
| rs33745945 | 46.4 | H | H | H | A | H | H | H | A | H | A | A | A | H | A | A | H | A | A | A | H | H | H | No |
| rs3699026 | 48.8 | H | H | H | H | H | H | H | A | A | A | A | A | H | H | A | H | A | A | A | H | H | H | No |
| rs30353028 | 50.5 | H | H | H | H | H | H | H | A | A | A | A | A | H | H | A | H | A | A | A | H | A | H | No |
| D9MIT97 | 50.7 | H | H | H | H | H | H | H | A | A | A | A | A | H | H | A | H | A | A | A | H | A | H | No |
| D9MIT971 | 50.8 | H | H | H | H | H | H | H | A | A | A | A | A | H | H | A | H | A | A | A | H | A | H | No |
| rs3699358 | 51.7 | H | H | H | H | H | H | H | A | A | A | A | A | H | H | A | H | A | A | A | H | A | H | No |
| rs6167828 | 51.9 | H | H | H | H | H | H | H | A | A | A | A | A | H | H | A | H | A | A | H | H | A | H | No |
| rs3685939 | 53.5 | H | H | H | H | H | H | H | A | A | A | A | A | H | H | A | H | A | A | H | H | A | H | No |
| rs29645267 | 53.9 | H | H | H | H | H | H | H | A | A | A | H | A | H | H | A | H | A | A | H | H | A | H | No |
| rs29835751 | 54.2 | H | H | H | H | H | H | H | A | A | A | H | A | H | H | A | H | A | A | H | H | A | H | No |
| rs30089733 | 54.3 | H | H | H | H | H | H | H | H | A | A | H | A | H | H | A | H | A | H | H | H | A | H | No |
| rs225183040 | 54.3 | H | H | H | H | H | H | H | H | A | A | H | A | H | H | A | H | A | H | H | H | A | H | No |
| rs29736523 | 54.3 | H | H | H | H | H | H | H | H | A | A | H | A | H | H | A | H | H | H | H | H | A | H | No |
| rs13480208 | 55.2 | H | H | H | H | H | H | H | H | A | H | H | A | H | H | A | H | H | H | H | H | A | H | No |
| rs30424912 | 56.7 | H | H | H | H | H | H | H | H | A | H | H | H | H | H | A | H | H | H | H | H | A | H | No |
| rs3677551 | 56.7 | H | H | H | H | H | H | H | H | A | H | H | H | H | H | H | H | H | H | H | H | A | H | No |
| rs29785790 | 56.7 | H | H | H | H | H | H | H | H | A | H | H | H | H | H | H | H | H | H | H | H | A | H | No |
| rs30437080 | 56.7 | H | H | H | H | H | H | H | H | A | H | H | H | H | H | H | H | H | H | H | H | A | H | No |
| rs30226504 | 56.7 | H | A | A | H | H | H | H | H | A | H | H | H | H | H | H | H | H | H | H | H | A | H | No |
| rs29946730 | 56.7 | H | A | A | H | H | H | A | A | A | A | A | H | H | H | H | H | H | H | H | H | A | H | No |
| rs4227682 | 57.3 | H | A | A | H | H | H | A | A | A | A | A | H | H | H | H | H | H | H | H | A | A | H | No |
| D9MIT21 | 57.7 | X | A | A | X | H | H | A | A | A | A | A | H | H | H | H | H | H | H | H | A | A | H | No |
| rs30042362 | 57.7 | A | A | A | A | H | H | A | A | A | A | A | H | H | H | H | H | H | H | H | A | A | H | No |
| rs29737283 | 57.9 | A | A | A | A | H | A | A | A | A | A | A | A | A | A | A | H | H | H | H | A | A | H | No |
| rs4227694 | 58.3 | A | A | A | A | H | A | A | A | A | A | A | A | A | A | A | H | H | H | H | A | A | H | No |
| rs3685575 | 59.4 | A | A | A | A | H | A | A | A | A | A | A | A | A | A | A | A | A | A | A | A | A | A | No |
| rs8254399 | 124.0 | A | A | A | A | H | A | A | A | A | A | A | A | A | A | A | A | A | A | A | A | A | A | No |
