# Supplementary material for: Adiposity QTL Adip20 decomposes into at least four loci when dissected using congenic strains
Source: PLoS One. 2017 Dec 1;12(12):e0188972. doi: 10.1371/journal.pone.0188972 (PMC5711020; doi:10.1371/journal.pone.0188972)
Supplement: S11 Table — Type = mapping population; Con = congenic; Overlap refers to QTL1- QTL4 as identified by the sequential method. Approach = method of estimating confidence interval (LDR = logarithmic transformed probability; LOD = logarithm of the odds); Ref = reference. (DOCX) [file pone.0188972.s011.docx]

| **QTL** | **Parental strains** | **Type** | **Peak, bp** | **CI, bp** | **Overlap** | **Approach** | **Ref** |
| --- | --- | --- | --- | --- | --- | --- | --- |
| *Adip5* | LG/J × SM/J | F_2_ | 76462664 | 76462846-120362686 | QTL4 | 1 LDR support | [1] |
| *Adip14* | SM/J × NZB/BIN | F_2_ | 109647579 | 86239845-120289624 | QTL4 | by adjacent markers | [2] |
| *Dob2* | SWR/J × AKR/J | F_2_ | 120289445 | 86239845-120289445 | QTL4 | by adjacent markers | [3] |
| *Mobq8* | C57BL/6J × CAST/Ei | F_2_ | 76462664 | 3739490-99910333 | QTL1-3 | by adjacent markers | [4] |
| *Obq5* | C57BL/6J × KK/HlLt | F_2_ | 41887849 | 28592037-41888030 | None | 1 LOD drop | [5] |
| *Carfhq2* | C57BL/6J × CAST/Ei | Con | 30000000 | 9100000-84200000 | QTL1-3 | CAST donor region | [6] |
| *plbcq5* | FVB/NJ × M16i | F_2_ | 62000000 | 35900000-125600000 | QTL1-4 | 1 LOD drop in cM | [7] |
| *Tdmq1* | BKS × DBA/2 | F_2_ | 46233355 | 40508298-51897370 | QTL1-2 | Bootstrap estimated | [8] |

Type=mapping population, Con=congenic, Overlap refers to QTL1 to 4 as identified in the accompanying text. Approach =method of estimating confidence interval. Ref=reference.

References

1. Cheverud JM, Vaughn TT, Pletscher LS, Peripato AC, Adams ES, Erikson CF, et al. Genetic architecture of adiposity in the cross of LG/J and SM/J inbred mice. Mamm Genome. 2001;12(1):3-12. PubMed PMID: 11178736.

2. Stylianou IM, Korstanje R, Li R, Sheehan S, Paigen B, Churchill GA. Quantitative trait locus analysis for obesity reveals multiple networks of interacting loci. Mamm Genome. 2006;17(1):22-36. PubMed PMID: 16416088.

3. West DB, Waguespack J, York B, Goudey-Lefevre J, Price RA. Genetics of dietary obesity in AKR/J x SWR/J mice: segregation of the trait and identification of a linked locus on chromosome 4. Mamm Genome. 1994;5(9):546-52. PubMed PMID: 8000138.

4. Mehrabian M, Wen PZ, Fisler J, Davis RC, Lusis AJ. Genetic loci controlling body fat, lipoprotein metabolism, and insulin levels in a multifactorial mouse model. J Clin Invest. 1998;101(11):2485-96. PubMed PMID: 9616220.

5. Taylor BA, Tarantino LM, Phillips SJ. Gender-influenced obesity QTLs identified in a cross involving the KK type II diabetes-prone mouse strain. Mamm Genome. 1999;10(10):963-8. PubMed PMID: 10501964.

6. Farber CR, FArber EA, De Vera V, Gluarte R, Castellani LW, Lusis AJ, et al., editors. Mouse chromosome 9 congenic-derived F2 intercrosses refine the location of a major obesity quantitative trait locus (QTL) (Carfhg2) and uncover a distinct QTL influencing plasma cholesterol (Cholhg1). Keystone; 2007; Keystone, Colorado.

7. Gordon RR, Hunter KW, Sorensen P, Pomp D. Genotype x diet interactions in mice predisposed to mammary cancer. I. Body weight and fat. Mamm Genome. 2008. PubMed PMID: 18286334.

8. Yaguchi H, Togawa K, Moritani M, Itakura M. Identification of candidate genes in the type 2 diabetes modifier locus using expression QTL. Genomics. 2005;85(5):591-9. Epub 2005/04/12. doi: 10.1016/j.ygeno.2005.01.006. PubMed PMID: 15820311.
