# Supplementary material for: Adiposity QTL Adip20 decomposes into at least four loci when dissected using congenic strains
Source: PLoS One. 2017 Dec 1;12(12):e0188972. doi: 10.1371/journal.pone.0188972 (PMC5711020; doi:10.1371/journal.pone.0188972)
Supplement: S12 Table — aThere are 277 genes within the donor region of strain 4 (42.6 to 58.3 Mb). Genes that passed the filters for differential expression in both Experiment 1 and 2 we display with blue font. Two genes in red font miss this criterion but their human orthologues are associated with adiposity or body mass index in GWAS studies (see Table 2). We show the remaining genes in black font. (DOCX) [file pone.0188972.s012.docx]

| **Experiment** | **Dot color** | **Absolute Log2FoldChange** | **FDR** | **Gene symbols** | **Percent (%)** |
| --- | --- | --- | --- | --- | --- |
| 1 | Green | >0.584963 | <0.05 | *Abcg4, Alg9, C230081A13Rik, Dixdc1, Fam55d, Hmbs, Il10ra, Pts, Sik2* | 3.2 |
|  | Red | <0.584963 | <0.05 | *1110032A03Rik, Apoc3, Arcn1, Cryab, Dlat, Dpagt1, Fxyd6, Gldn, Kdelc2, Mcam, Neil1, Pafah1b2, Pdzd3, Sidt2, Tmprss5, Ube4a* | 5.8 |
|  | Orange | >0.584963 | >0.05 | *Acsbg1, Apoa1, Bco2, Ccdc84, Cd3g, Fam55b, Hmg20a, Isl2, Mpzl2, Mpzl3, *Ncam1, Nnmt, Pou2af1, Ptpn9, Sik3* | *5.4* |
|  | Black | <0.584963 | >0.05 | rest of the 237 genes^a^ | 85.6 |
| 2 | Green | >0.584963 | <0.05 | *Abcg4, Dixdc1, Fam55d, Il10ra, Mpzl3, Sh2d7, Sik2* | 2.5 |
|  | Red | <0.584963 | <0.05 | *Arcn1, Cep164, Chrnb4, Cryab, naja4, *Fdx1, Fxyd6, Hmbs, Il18, Mcam, Oaf, Ptpn9, Rexo2, Usp2, Vps11* | 5.4 |
|  | Orange | >0.584963 | >0.05 | *1600029D21Rik, Acsbg1, AI118078, Alg9, Bud13, C230081A13Rik, Cd3g, Crabp1, Drd2, Fxyd2, Isl2, Mpzl2, Nnmt, Scn2b, Sgip1, Sik3, Sln* | 6.1 |
|  | Black | <0.584963 | >0.05 | rest of the 238 genes^a^ | 85.9 |
| Reproducible genes | Red text labeled points | Filter applied FDR < 0.05 and/or absolute log2FoldChange > 0.584963 | | *Abcg4, Acsbg1, Alg9, Arcn1, Cd3g, Cryab, C230081A13Rik, Dixdc1, Fxyd6, Fam55d, Hmbs, Il10ra, Isl2, Mcam, Mpzl2, Mpzl3, Nnmt, Ptpn9, Sik2, Sik3* | 7.2 |
