# Supplementary material for: Adiposity QTL Adip20 decomposes into at least four loci when dissected using congenic strains
Source: PLoS One. 2017 Dec 1;12(12):e0188972. doi: 10.1371/journal.pone.0188972 (PMC5711020; doi:10.1371/journal.pone.0188972)
Supplement: S1 Text — Explanation for each of the three branches of the minimum spanning tree (MST; central, left, and right) in Fig 3. Arrows after QTLs indicate the direction of effect of the 129-derived allele. (DOC) [file pone.0188972.s013.doc]

**Supplemental Text 1**. Logic of the sequential method for each of the three branches of the minimum spanning tree (MST; central, left, and right) in **Figure 3.** Arrows after QTLs indicate the direction of effect of the 129-derived allele.

Analysis of the central branch of the MST supports the presence of QTL1↑, QTL2↑, and QTL3↓. Mice from strain 3.1.1.1 differed by host-donor genotype, which indicates the presence of QTL3↓. Strain 3.1.1.4 has similar adipose depot weight compared to strain 3.1.1.1, indicating it does not contain any additional QTLs besides QTL3↓. Mice from strain 3.1.1 have heavier gonadal adipose depot weights on average compared with strain 3.1.1.4, which indicates that strain 3.1.1 harbors QTL2↑. QTL3↓ is present in both these strains (3.1.1.4 and 3.1.1). Mice from strain 1.1 have heavier adipose depot weight on average compared with those from strain 3.1.1, which indicates that strain 1.1 harbors QTL1↑ in addition to QTL2↑ and QTL3↓. Mice from strain 1 and strain 1.1 have similar average gonadal adipose depot weights, indicating the donor regions from these strains do not contain any QTLs in addition to QTL1↑, QTL2↑, and QTL3↓.

Analysis of the left branch of the MST confirms QTL1↑. We draw this conclusion because strain 3.1.1.2 does not differ in gonadal adipose depot weight compared with strain 3.1.1.2. Strain 4.4 has heavier gonadal depot weights on average compared with strain 3.1.1.2, consistent with the presence of QTL1↑ in strain 4.4 that is not present in strain 3.1.1.2.

Analysis of the right branch of the MST confirms QTL4↑ in addition to confirming QTL1↑, QTL2↑, and QTL3↓. Neither strain 3.1.4.1 nor strain 4.1 differed in gonadal adipose depot weight when we compared host and donor genotype; hence, these two strains do not harbor QTLs. Strain 3.1 has a heaver adipose depot weight compared with strain 4.1, which is consistent with a combined effect of QTL2↑ and QTL3↓. Strain 4 has heavier adipose depot weight than strain 3.1, which indicates that strain 4 harbors QTL1↑ in addition to QTL2↑ and QTL3↓. Mice with the donor region from strains 3 and 4 have gonadal depots of the same weight even though strain 3 does not harbor QTL1↑; to account for this result, we infer the presence of QTL4↑, which strain 4 has but strain 3 does not.
