# Supplementary figures and images for: Adiposity QTL Adip20 decomposes into at least four loci when dissected using congenic strains
Source: PLoS One. 2017 Dec 1;12(12):e0188972. doi: 10.1371/journal.pone.0188972 (PMC5711020; doi:10.1371/journal.pone.0188972)

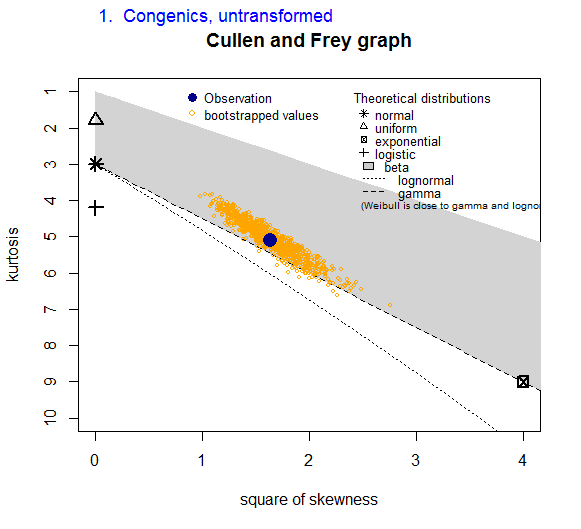

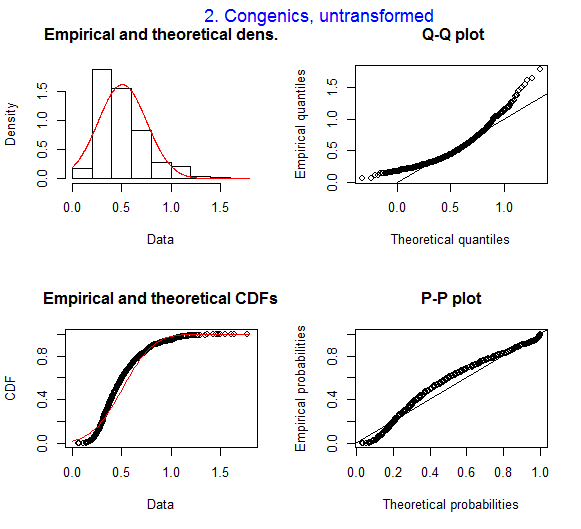

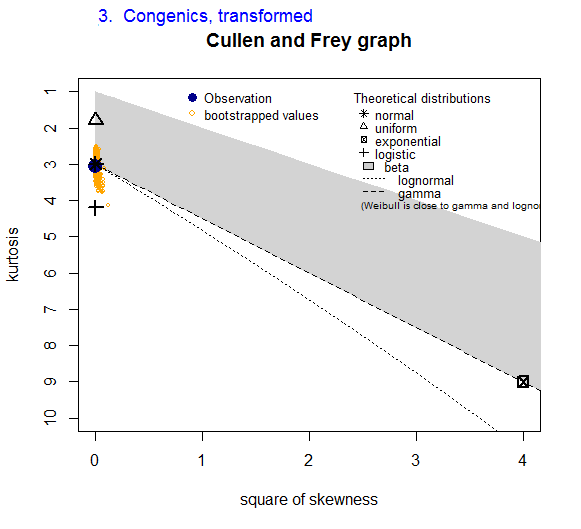

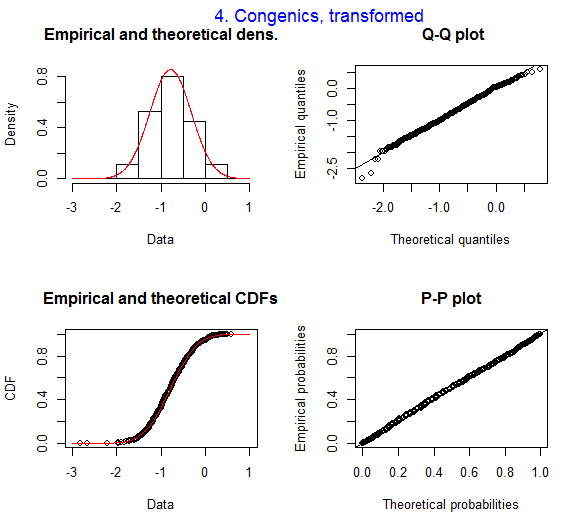

Supplement: S1 Fig — Cullen and Frey graphs and empirical and theoretical data for untransformed and log-transformed congenics data. We tested the distribution of the gonadal adipose depot weight data for normality; S3 Table contains the parameters of the model-fitting untransformed and log-transformed data. CDF = cumulative distribution function. (DOCX) [file pone.0188972.s014.docx]

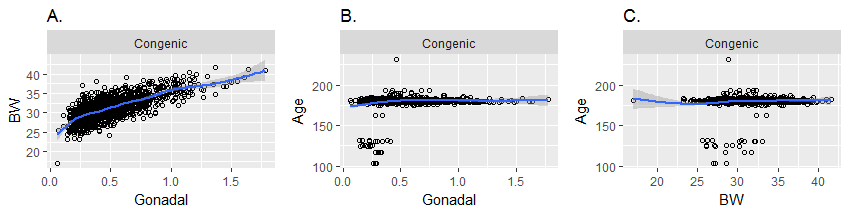

Supplement: S2 Fig — (A) gonadal adipose depot and body weight, (B) gonadal adipose depot and age as well as (C) body weight and age of all congenic mice. Gonadal = Gonadal adipose depot in grams; BW = body weight in grams; Age in days. (DOCX) [file pone.0188972.s015.docx]

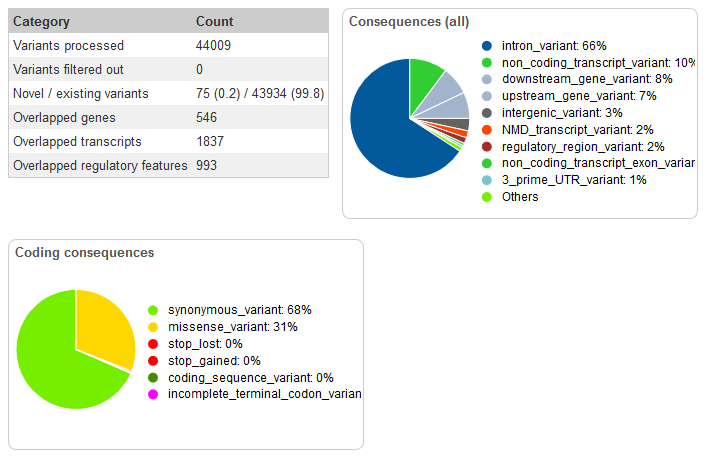

Supplement: S3 Fig — In total, we uploaded 44,009 variants for the variant function analyses to the online tool Variant Effect Predictor; we found data from nearly all variants (99.8%) using this tool; of these variants, 2% were regulatory variants, and 31% of variants in coding regions were missense variants. (DOCX) [file pone.0188972.s016.docx]

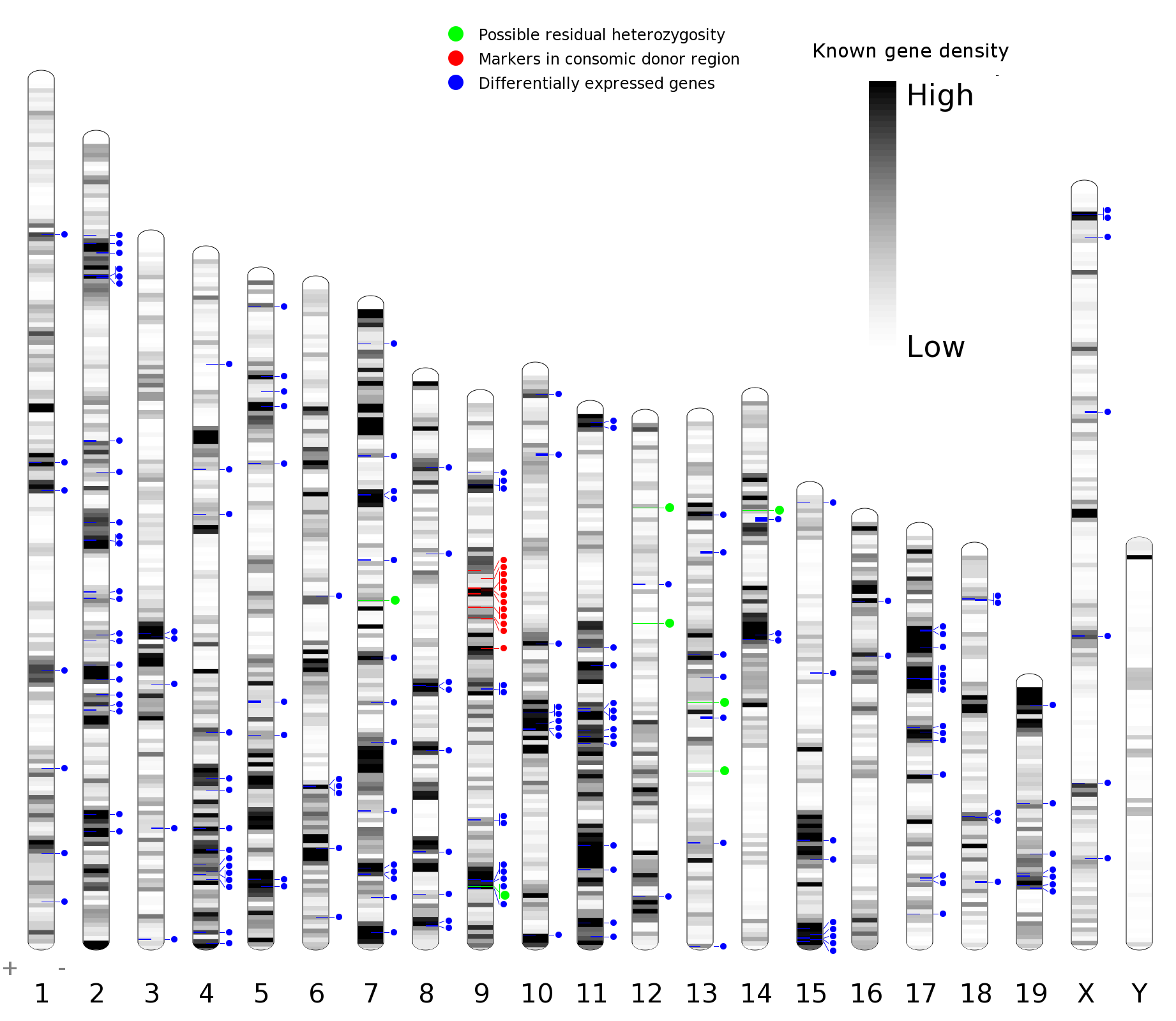

Supplement: S4 Fig — Genome-wide overview of the microarray data from Experiment 1 with differentially expressed genes (blue dots) in adipose tissue between genotypes (129/B6 vs B6/B6) in congenic male mice. We observed potential heterozygosity (green dots) of this congenic strain in at least one of the samples at 25 of the 2,715 polymorphic markers. Of these, 19 were located in the congenic donor region (red dots), leaving 7 markers (green dots) with potential residual heterozygosity that may have produced differential gene expression independent of the donor region. (DOCX) [file pone.0188972.s017.docx]
